# A Comparison of Cytotoxicity and Oxidative Stress from Welding Fumes Generated with a New Nickel-, Copper-Based Consumable versus Mild and Stainless Steel-Based Welding in RAW 264.7 Mouse Macrophages

**DOI:** 10.1371/journal.pone.0101310

**Published:** 2014-06-30

**Authors:** Melissa A. Badding, Natalie R. Fix, James M. Antonini, Stephen S. Leonard

**Affiliations:** Health Effects Laboratory Division, National Institute for Occupational Safety and Health, Morgantown, West Virginia, United States of America; Taipei Medical University, Taiwan

## Abstract

Welding processes that generate fumes containing toxic metals, such as hexavalent chromium (Cr(VI)), manganese (Mn), and nickel (Ni), have been implicated in lung injury, inflammation, and lung tumor promotion in animal models. While federal regulations have reduced permissible worker exposure limits to Cr(VI), this is not always practical considering that welders may work in confined spaces and exhaust ventilation may be ineffective. Thus, there has been a recent initiative to minimize the potentially hazardous components in welding materials by developing new consumables containing much less Cr(VI) and Mn. A new nickel (Ni) and copper (Cu)-based material (Ni-Cu WF) is being suggested as a safer alternative to stainless steel consumables; however, its adverse cellular effects have not been studied. This study compared the cytotoxic effects of the newly developed Ni-Cu WF with two well-characterized welding fumes, collected from gas metal arc welding using mild steel (GMA-MS) or stainless steel (GMA-SS) electrodes. RAW 264.7 mouse macrophages were exposed to the three welding fumes at two doses (50 µg/ml and 250 µg/ml) for up to 24 hours. Cell viability, reactive oxygen species (ROS) production, phagocytic function, and cytokine production were examined. The GMA-MS and GMA-SS samples were found to be more reactive in terms of ROS production compared to the Ni-Cu WF. However, the fumes from this new material were more cytotoxic, inducing cell death and mitochondrial dysfunction at a lower dose. Additionally, pre-treatment with Ni-Cu WF particles impaired the ability of cells to phagocytize *E. coli*, suggesting macrophage dysfunction. Thus, the toxic cellular responses to welding fumes are largely due to the metal composition. The results also suggest that reducing Cr(VI) and Mn in the generated fume by increasing the concentration of other metals (e.g., Ni, Cu) may not necessarily improve welder safety.

## Introduction

Epidemiology studies have provided strong evidence of welders developing respiratory illness due to their workplace exposures (reviewed in [1]). This includes occupational asthma [2], bronchitis [3], and lung cancer [4], [5]. These diseases arise from the inhalation of vaporized particulate matter generated during welding processes. High temperatures and a consumable electrode are used to join base metal pieces, forming fumes that contain oxidized metals which originate from the consumable electrode wire (reviewed in [1]).

Some of these aerosolized metals from stainless steel (SS) welding are known to be toxic and carcinogenic, such as chromium (Cr), manganese (Mn), and nickel (Ni). Previous studies have strongly implicated Cr(VI) in the production of damaging reactive oxygen species (ROS), pulmonary injury, and carcinogenesis following SS welding fume lung exposures [6]–[8]. Inhalation of Mn-containing fumes are associated with neurological issues such as early-onset Parkinsonism ([9], reviewed in [10], [11]) in addition to pulmonary effects [12]. The carcinogenic potential of Ni content in welding fumes has raised concerns as well [13], [14]. Therefore, it seems advantageous to alter the components of welding consumables in order to reduce the potential toxicities from fume exposures. However, when the metallic makeup of an electrode is changed, its “weldability” and other physical properties can be altered in an unfavorable way.

A newly developed welding material, which is a Ni- and copper (Cu)-based alloy, is being proposed as an alternative to SS consumables due to its minimal Cr, iron (Fe), and Mn content. Tests of the mechanical properties and weldability indicate that it performs similarly to SS, but produces significantly lower levels of airborne Cr(VI) compared to SS welding [15]. These factors make for an attractive potential substitute for steel-based alloys used in the welding industry. However, fumes from this new material (termed Ni-Cu WF in our study) do contain potentially toxic metals, so an analysis of cellular responses to Ni-Cu WF needs to be performed. Fumes generated by gas metal arc welding with mild steel (GMA-MS) and stainless steel (GMA-SS) have been previously examined in several *in vitro* and *in vivo* toxicity studies by our group and others. Thus, these materials can provide helpful comparisons with the new Ni-Cu WF particles in terms of toxicity assessments.

Although this new Ni-Cu-based consumable contains drastically less Cr(VI), Fe, and Mn yet retains favorable welding properties similar to SS welding, there has been no toxicological testing to evaluate whether it may be less hazardous for exposed welders. Therefore, the main goal of the current study was to determine if this welding fume causes adverse cellular responses and to examine how its cytotoxicity compares to previously-studied steel-based fumes which are known to be toxic.

## Materials and Methods

### Welding materials

Two gas metal arc welding fume samples were kindly generated and provided by Lincoln Electric Co. (Cleveland, OH). Bulk samples of the fumes were generated in a cubical open front fume chamber (volume = 1 m^3^) by a skilled welder using a semi-automatic technique appropriate to the electrode and collected on 0.2 µm Nuclepore filters (Nuclepore Co., Pleasanton, CA). The two fume samples were generated in following ways: (1) gas metal arc welding using a mild steel E70S-3 electrode (GMA-MS) and (2) gas metal arc welding using a stainless steel ER308L Si electrode (GMA-SS) with argon and CO_2_ shielding gases to protect the weld from oxidation. The welding fume generated from the Ni-Cu-based consumable (Ni-Cu WF) was produced by the Welding and Joining Metallurgy Group at The Ohio State University (Columbus, OH) during shielded metal arc (SMA) welding using a consumable with a target composition of Ni-7.5Cu-1Ru that contained other alloying agents. The fume samples were collected onto electrostatic filter medium (PE 1306NA; Hollingsworth and Vose, East Walpole, MA). For cellular exposure experiments, particles were suspended in sterile filtered 1X phosphate-buffered saline (PBS) at a 1 mg/ml stock, vortexed, and diluted into cell culture media at a low (50 µg/ml) or high dose (250 µg/ml), based on previous *in vitro* studies of GMA-MS and GMA-SS fumes [7], [16]. Welding fume stocks were prepared freshly for experiments. Equal volumes of 1X PBS were used as control conditions for each experiment.

### Particle size and elemental analysis

Particle sizes of the welding samples collected onto filters in bulk were characterized by scanning electron microscopy and all three were of respirable size with count mean diameters of <2 µm. Replicate welding fume samples were analyzed for Cr(VI) levels using NIOSH method 7605 [17]. Briefly, 5 ml of extraction solution (3% Na_2_CO_3_/2% NaOH) were added to each 5 mg sample, and the tubes were sonicated in a bath for 30 min. This procedure extracts both soluble and insoluble Cr(VI) present in the fumes. Analysis used a Dionex HPIC-AS7 column with 250 mM (NH_4_)_2_SO_4_/100 mM NH_4_OH mobile phase and a post-column reagent (2.0 mM diphenylcarbazide/10% methanol/1N H_2_SO_4_) with absorbance detection at 540 nm. Four concentrations of standards were made from a certified Cr(VI) solution, covering a range of 0.4–4 µg/ml. The estimated limit of detection is 0.02 µg, and the method range is 0.05 to 20 µg of Cr(VI). Analysis of other elements present in the welding fume samples was done using inductively coupled plasma-atomic emission spectroscopy (ICP-AES) using NIOSH method 7300 modified for microwave digestion (NIOSH 1994).

In addition, portions of the different welding fume samples (GMA-SS, GMA-MS, and Ni-Cu WF) were suspended in distilled water, pH 7.4, and sonicated for 1 min with a Sonifier 450 Cell Disruptor (Branson Ultrasonics, Danbury, CT, USA) to determine particle/metal solubility. The three particle suspensions (total samples) were incubated for 24 hours at 37°C, and the samples were centrifuged at 12,000 g for 30 min. The supernatants of the samples (soluble fraction) were recovered and filtered with a 0.22 µm filter (Millipore Corp., Bedford, MA, USA). The pellets (insoluble fraction) were resuspended in water. The sample suspensions (total, soluble, and insoluble fractions) were digested, and the metals analyzed by ICP-AES according to NIOSH method 7300 [18]. All three welding fumes were observed to be relatively insoluble with soluble/insoluble ratios of 0.006, 0.020, and 0.004 for the GMA-MS, GMA-SS, and Ni-Cu WF samples, respectively.

### Cell culture

The adherent mouse monocyte-derived macrophage cell line, RAW 264.7, was obtained from American Type Culture Collection (Manassas, VA). The cells were cultured in Dulbecco’s modified Eagle’s medium (DMEM) with 2 mM L-glutamine, 10% fetal bovine serum, and 50 mg/ml penicillin/streptomycin (Invitrogen Life Sciences, Grand Island, NY). Cells were grown at 37°C in a 5% CO_2_ incubator and passaged by scraping into medium or PBS, depending on the experiment.

### Viability assays

Cells were plated at 2×10^5^ cells/well in 24-well dishes, treated with suspensions of welding fumes (50 µg/ml or 250 µg/ml), and incubated at 37°C for 24 hours. Wells were washed twice with 1X PBS, scraped into 250 µl PBS, mixed 1∶1 with trypan blue (vol:vol) and cell counts were calculated using a Countess Automated Cell Counter (Life Technologies). Data from 4 independent experiments are reported as the mean number of viable cells and percent viability out the cell population for each condition.

For viability measurements in the presence of an antioxidant, cells were plated at 1×10^5^ cells/ml in 96-well dishes, and half the wells were pre-treated with 20 mM *N*-Acetyl-L-cysteine (Sigma-Aldrich, St. Louis, MO) for 1 hour prior to treatments with suspensions of welding fumes. Changes in cell viability at 24 hours were assessed using the MultiTox-Fluor Cytotoxicity Assay according to the manufacturer’s directions (Promega, Madison, WI). A cell-permeable protease substrate, GF-AFC (glycyl-phenylalanyl-amino-fluorocoumerin), is cleaved by live cells to produce fluorescent AFC. Thus, the fluorescent signal is proportional to the number of viable cells. To ensure the fluorescent signal was due to substrate products and not autofluorescence or interference by the welding fume suspensions, separate wells of medium and fume suspensions were included in the plates, and these readings were subtracted from their respective wells that contained treated cells. All conditions were run in triplicate wells and three independent experiments were performed.

### Mitochondrial oxygen consumption

Cells were plated at 12,500 cells/well in 96-well Seahorse XFe-96 assay plates for 24 hours before being treated with welding fume suspensions (50 µg/ml or 250 µg/ml) for another 24 hours. On the day of metabolic flux analysis, cells were changed to unbuffered DMEM media (DMEM supplemented with 25 mM glucose, 10 mM sodium pyruvate; pH 7.4) and incubated at 37°C in a non-CO_2_ incubator for 1 hour. All media was adjusted to pH 7.4 on the day of assay. Six baseline measurements of oxygen consumption rate (OCR) were taken before sequential injection of mitochondrial inhibitors, oligomycin (10 µM), FCCP (1 µM; carbonilcyanide *p*-triflouromethoxyphenylhydrazone) and rotenone (5 µM). Four measurements were taken after each addition of mitochondrial inhibitor before injection of the next inhibitor. Five independent experiments were performed. Oxygen consumption rate, an indicator of oxidative phosphorylation, was automatically calculated and recorded by the Seahorse XFe-96 software (Seahorse Bioscience, North Billerica, MA, USA).

### Electron spin resonance

To detect and measure short-lived free radical intermediates, electron spin resonance (ESR) spin-trapping was used. To assess whether the welding fume suspensions are capable of producing hydroxyl radicals (•OH) after exposure to H_2_O_2_ through a Fenton-like reaction, final concentrations of 1 mg/ml compounds, 1 mM H_2_O_2_, and 100 mM DMPO spin trap (5,5′-dimethylpyrroline *N*-oxide, Sigma-Aldrich) were mixed in test tubes for 3 min at room temperature and transferred to a quartz flat cell for ESR measurement in a Bruker EMX spectrometer (Bruker Instruments Inc., Billerica, MA). For each sample, the instrument was set to run 10 scans with a 41 sec scan time, a receiver gain of 2.5×10^4^, a 40 msec time constant, 1.0 G modulation amplitude, 126.9 mW power, 9.751 frequency, and 3475±100 G magnetic field. Signal intensity (peak height) from the 1∶2∶2∶1 spectra, which is characteristic of •OH [19] was used to measure the relative amount of short-lived radicals trapped.

For cellular ESR, final concentrations of 2×10^6^ RAW 264.7 cells/ml, 1 mg/ml welding fume suspensions, and 200 mM DMPO in PBS were mixed in test tubes. Experiments were carried out in the presence and absence of the H_2_O_2_ scavenger catalase (2000 U/ml), as previously described [20]–[22]. Samples were incubated at 37°C for 5 min and were loaded into a flat cell and scanned as in previous cellular ESR experiments [20], [23]–[25]. Again, peak heights represent relative levels of trapped hydroxyl radicals.

### Intracellular ROS (DCFH assay)

Cells were plated at 1×10^5^ cells/ml in 96-well dishes and treated with the cell-permeable fluorogenic probe DCFH-DA (2′,7′-dichlorodihydrofluorescein diacetate, Cell Biolabs, Inc., San Diego, CA) at a final concentration of 1 mM in serum-free DMEM for 45 min at 37°C. Cells were washed twice with 1X PBS and DMEM was added back to the wells, along with 50 µg/ml welding fume suspensions. Upon ROS production, DCFH-DA is oxidized to form DCF, which is highly fluorescent. The cells were incubated at 37°C for 7 hours, and plates were read at 485 nm excitation/530 nm emission each hour to measure any change in fluorescence, indicating ROS production. To ensure the fluorescent signal was due to DCF product and not any autofluorescence that may emit from the welding fume suspensions, separate wells of DMEM and each fume were included in the plates, and these readings were subtracted from their respective wells that contained treated cells. All conditions were run in triplicate wells and three independent experiments were performed.

### Comet assay

Cells were plated at 2×10^5^ cells/ml in 24 well plates, treated with or without 50 µg/ml welding fume suspensions for 3 hours, washed twice and scraped into 1X PBS, added to glass slides with agarose, then lysed and subjected to electrophoresis according to manufacturer’s instructions (Trevigen Inc., Gaithersburg, MD). This causes fragmented DNA to migrate out of the nuclear region, forming a comet-like tail, which was labeled with SYBR green (binds double-stranded DNA). Images were acquired using an Olympus AX70 microscope equipped with an Olympus DP73 digital camera (Olympus, Center Valley, PA). Two independent experiments were performed and at least 50 total cell comets per condition (23–30 comets per replicate) were measured for the percentage of DNA in comet tails per condition. This was calculated by determining the background-corrected fluorescence from: the nuclear region of interest in a cell (nuclear ROI) and the total cell fluorescence for a defined region of interest (total cell ROI). ImageJ software [26] was used to measure the “integrated density” (the sum of the values of the pixels in the selection) for regions of interest, and Microsoft Excel was used to calculate the corrected fluorescence values and ratios of nuclear ROI to total cell ROI. This was converted to a percentage of DNA in the “tail” (100 – [(nuclear ROI/total cell ROI)×100]), with elevated percentages indicating DNA damage.

### Phagocytosis assay

Cells were plated at 1.5×10^5^ cells/ml in 96-well dishes and pre-treated with 50 µg/ml welding fume suspensions for 3 or 6 hours prior to the addition of pHrodo Red *E. coli* BioParticles, which were prepared according to the manufacturer’s instructions (Molecular Probes, Carlsbad, CA). Briefly, *E. coli* BioParticles were suspended in Live Cell Imaging Solution at 1 mg/ml and sonicated for 5 min. At each time point, wells were washed twice with warm Live Cell Imaging Solution, and 100 µl of suspended BioParticles were added to all wells, including blanks (no cells) to be subtracted from all wells. Following 2 hours incubation at 37°C, plates were read at 560 nm excitation/600 nm emission in a plate reader to measure changes in fluorescence. Cytochalasin D (Sigma-Aldrich) was added to triplicate wells 1 hour prior to the end of each time point as a positive control for preventing phagocytosis. It was added back to those wells with the BioParticles at a final concentration of 10 µM, allowing 3 hours total incubation with the phagocytosis inhibitor. All conditions were run in triplicate wells and four independent experiments were performed. The percentage phagocytosis was calculated as a ratio of the net experimental phagocytosis to the untreated control cell phagocytosis.

### Enzyme-linked immunosorbent assay (ELISA)

Cells were plated at 5×10^5^ cells/ml in 12-well dishes and treated with 50 µg/ml or 250 µg/ml welding suspensions for 24 hours. Lipopolysaccharide (LPS) at 1 µg/ml was used as a positive control (from *E. coli* 055:B5, Sigma-Aldrich Corp., St. Louis, MO). Media were collected from wells at the time points and frozen at −80°C. Cytokine levels were measured via BD OptEIA ELISA kits (mouse TNFα; cat. no. 555268, mouse IL-6; cat. no. 555240, mouse IL-1β; cat. no. 559603, BD Biosciences, San Diego, CA) according to manufacturer’s directions.

### Statistical analysis

All data are represented as the mean ± standard deviation (SD). A one-way or two-way analysis of variance (ANOVA) with a Tukey post-test was performed using GraphPad Prism 6 software (GraphPad Software, Inc., La Jolla, CA) for each experiment to compare the responses between groups, and statistical significance is shown when p<0.05. For the mitochondrial stress assay results, data were first log-transformed prior to analysis.

## Results

### Welding fume metal analysis

The welding fumes used in this study were generated from either gas metal arc welding with mild steel (GMA-MS) or stainless steel (GMA-SS) materials or by shielded metal arc welding using a nickel-copper-based consumable (Ni-Cu WF). **Table 1** lists the three welding samples and their respective metal content. The GMA-MS consisted mostly of Fe (82.8%) and Mn (15.2%), while GMA-SS had similar Mn content (13.8%) but less Fe (57.2%) and a large percentage of Cr (20.3%). The newly-developed Ni-Cu WF had a very different metal profile compared to the GMA-MS and GMA-SS fumes, as it contained very low levels of Fe, Mn, and total Cr. This welding fume contained mostly potassium (29.9%), aluminum (20.7%), Ni (13.4%), and titanium (11.7%). All three particles were similar in size and in the respirable range (<2 µm count mean diameters), and tests of particle and metal solubility showed all samples to be relatively insoluble in water. Therefore, any observed differences are likely to be due to the metal profiles of the fume samples.

**Table 1:** Metal content analysis of the welding fumes.

Welding Fume Samples	Elements (weight %)^a^	Cr(VI) (µg/g ± SE)
**GMA-MS**	**Iron 82.8%**	not detected
	**Manganese 15.2%**	
	Copper 1.84%	
	Aluminum 0.17%	
**GMA-SS**	**Iron 57.2%**	2600±120
	**Chromium 20.3%**	
	**Manganese 13.8%**	
	**Nickel 8.51%**	
	Copper 0.16%	
	Aluminum 0.10%	
**Ni-Cu WF**	Potassium 29.9%	422±35
	Aluminum 20.7%	
	**Nickel 13.4%**	
	Titanium 11.7%	
	Strontium 7.58%	
	Copper 6.00%	
	Magnesium 3.86%	
	Calcium 2.99%	
	**Iron 1.65%**	
	Phosphorus 0.65%	
	**Chromium 0.47%**	
	**Manganese 0.46%**	
	Barium 0.26%	
	Zinc 0.20%	
	Trace elements (<0.1 wt%): cobalt, lead, lithium, ruthenium, vanadium, zirconium	

a Relative to all metals analyzed; **bold** used to emphasize elements with the most toxic potential based on previous studies of welding fumes; SE, standard error.

### Welding fumes reduce cell viability

**Figure 1:**
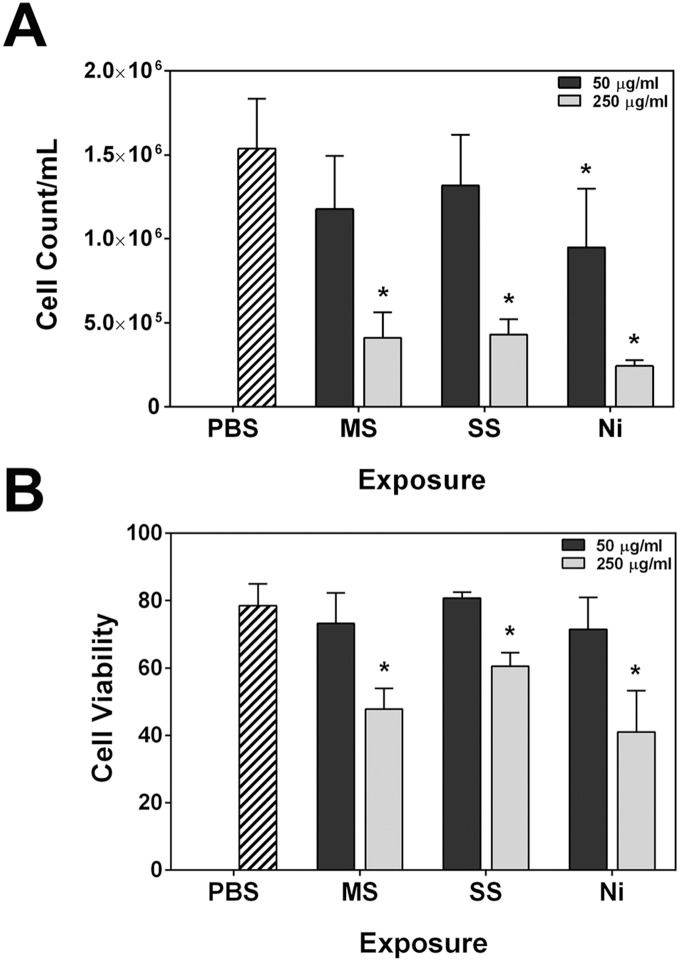
Welding fumes reduce RAW 264.7 cell viability. Cells were treated with suspensions of welding fumes for 24(**A**) and percentage of viable cells (**B**) within each sample. Error bars represent the mean ± SD (n = 4). *, p<0.05 compared to PBS control. MS; GMA-MS, SS; GMA-SS, Ni; Ni-Cu WF.

To determine the degree of welding fume cytotoxicity, viability assays were carried out in RAW 264.7 cells following 24 hour treatments with 50 µg/ml and 250 µg/ml doses. All three welding samples at the higher dose caused significant reductions in the concentration of live cells and overall viability using trypan blue exclusion (**Figure 1**). However, only treatment with Ni-Cu WF caused a significant reduction in the live cell concentration at the 50 µg/ml dose (9.5×10^5^ cells/ml, Ni-Cu WF vs. 1.5×10^6^ cells/ml, PBS), suggesting that this newly-developed welding material is more cytotoxic to RAW 264.7 macrophages than the GMA-MS and SS fumes.

**Figure 2:**
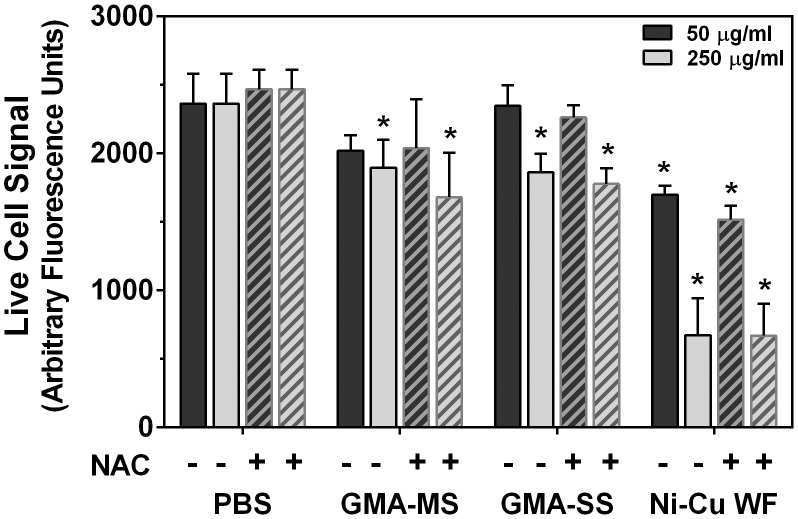
Reduced viability is not due to excessive ROS production. RAW 264.7 cells were pre-treated with and without *N*-acetyl-L-cysteine (NAC) for 1 h and then for 24 h with suspensions of welding fumes. MultiTox-Fluor Reagent (Promega) was added to all wells for 1 h at 37°C, and plates were read at 400ex/505em to determine the fluorescence signal from live cells. Error bars represent the mean ± SD (n = 3). There was no significant difference between − and + NAC for treatment groups. *, p<0.05 compared to PBS control.

Further, an additional cytotoxicity assay was performed to measure the activity of a protease that is only active in viable cells. In this assay, cells were pre-treated with or without the antioxidant *N*-acetyl-L-cysteine (NAC) to assess whether the observed reduced cell viability was due to excessive ROS production. This appeared to not be the case, as there were no significant differences between wells treated with and without NAC for the same welding fume dose (**Figure 2**). However, compared to PBS controls, there were significant reductions in the live cell signal at the 250 µg/ml dose, confirming cytotoxicity for all three welding fumes. Also in agreement with **Figure 1** results, only Ni-Cu WF induced significant reductions at the 50 µg/ml dose.

### Changes in bioenergetics and mitochondrial function following welding fume exposures

**Figure 3:**
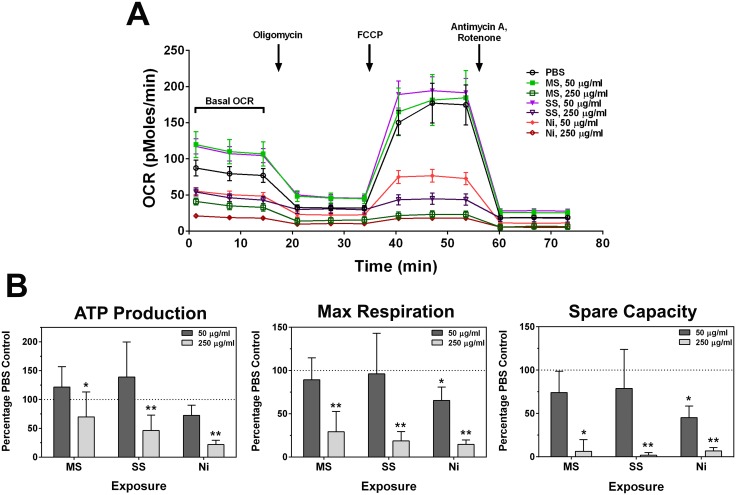
Mitochondrial dysfunction following incubations with welding fumes. RAW 264.7 cells were treated with suspensions of welding fumes. Following 24°C, media were removed and wells were washed 3 times with unbuffered XF media, and the plate was run using the mitochondrial stress test kit (Seahorse Biosciences) to determine the oxygen consumption rate (OCR) at certain time points. **A.** Representative mean OCR traces at baseline and following injections of reagents. **B.** ATP production, maximum respiration, and spare capacity were calculated from the mean OCRs from 5 independent experiments. Error bars represent the mean ± SD (n = 5). *, p<0.05 and **, p<0.0001 compared to PBS control.

As an additional approach to examine welding fume cytotoxicity and effects on viability, we measured mitochondrial function. Using the Seahorse Biosciences Extracellular Flux (XF) Analyzer, real-time changes in cellular bioenergetics were measured via a mitochondrial stress test. **Figure 3A** shows oxygen consumption rate (OCR) traces after 24 hour exposures to the welding fume samples from a representative experiment. We found that 50 µg/ml GMA-MS- and GMA-SS-treated cells in general had slightly higher basal OCRs than PBS-treated controls. Following a period of basal readings, compounds used to estimate bioenergetic function were added to the wells. Oligomycin inhibits the ATP synthase (Complex V) and was used to determine the extent by which OCR is linked to ATP production, FCCP is a proton uncoupler that forces maximal respiratory capacity, and antimycin A/rotenone inhibit Complexes III and I, respectively, to dramatically suppress the OCR. The resulting rates were used to calculate ATP production, maximum respiration, and spare capacity compared to control cells. Reductions in these parameters at the 250 µg/ml dose confirm the loss of viability induced by all three fumes as in **Figures 1** **and** **2**.

Pre-treatment of cells with 50 µg/ml Ni-Cu WF not only caused lower basal OCRs, but also prompted changes in the way mitochondria were able to deal with pharmacological inhibitors (**Figure 3B**). For example, following treatment with FCCP, the OCR spiked to provide maximal respiration, but this response was mitigated in cells pre-treated with Ni-Cu WF (65.4±15.4% of PBS control, mean ± SD). Whether this was due to fewer mitochondria because of proceeding cell death or actual mitochondrial dysfunction remains unclear; however, it is likely a combination of both factors that led to these results. Although it was not statistically significant for GMA-MS and GMA-SS, the reserve or spare capacity (maximum OCR – basal rate) was reduced compared to controls following 50 µg/ml treatments with all three fume samples (74.1±24.6% GMA-MS, 78.9±45.0% GMA-SS, and 45.1±13.5% Ni-Cu WF, mean ± SD). This measurement represents the respiratory capacity available for cells when responding to various stressors that increase bioenergetic demands. Its decrease has been implicated in oxidative stress conditions [27], [28], which may be induced by these welding fumes.

### Free radical production from welding fumes

**Figure 4:**
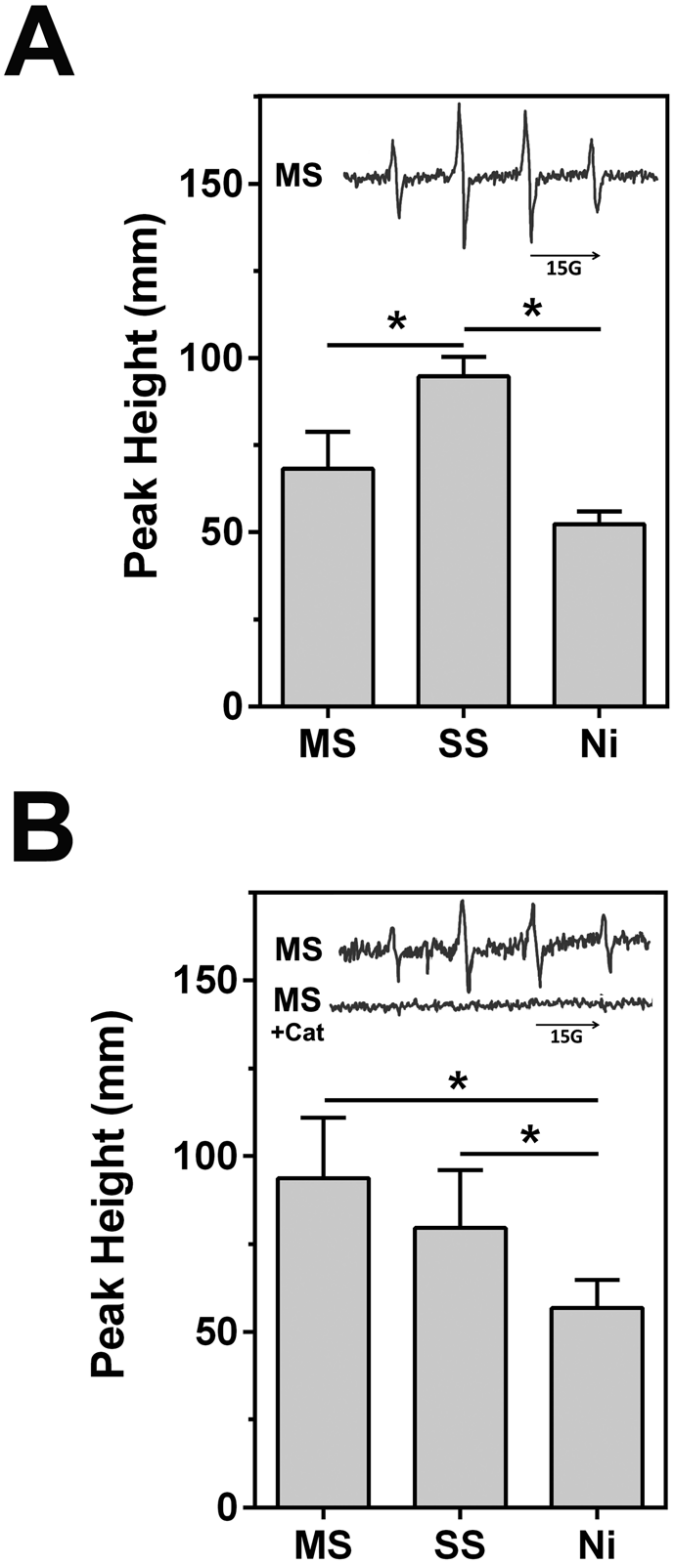
Electron spin resonance measurement of •OH radicals following reaction of welding fumes with H_2_O_2_ or RAW 264.7 cells. **A.** An acellular mixture of welding fume suspensions (1 mg/ml), H_2_O_2_ (1 mM), and spin trap DMPO (100 mM) in PBS were incubated in test tubes for 3 min at room temperature, and scanned via ESR. Signal intensity (peak height) is used to measure the relative amount of hydroxyl radicals and error bars represent the mean ± SD (n = 3). Inset: representative GMA-MS spectra. **B.** The same as in **A**, except without H_2_O_2_ but including 2×10^6^ RAW 264.7 cells/ml. Samples were incubated at 37°C for 5 min prior to measurements. (n = 5–6). Insets: representative GMA-MS spectra and with catalase treatment (+Cat) for cellular ESR. *, p<0.05.

Due to the previously-documented evidence of ROS production from welding fumes [7], [8], [16], [29] and effects on mitochondrial bioenergetics, the samples were tested for their ability to generate hydroxyl radicals via electron spin resonance (ESR) using spin trapping with DMPO (5,5′-dimethylpyrroline *N*-oxide). The acellular Fenton-like reactions were carried out with 1 mM H_2_O_2_ and 1 mg/ml welding fume suspensions to determine if the particles have the potential to convert H_2_O_2_ into •OH as in previous studies [16], [24], [30]. Each of the samples induced ESR peaks in a 1∶2∶2∶1 spectra, indicating •OH radical generation [19], but the GMA-SS fume produced significantly higher peaks compared to the other two samples (94.7±5.6 mm GMA-SS vs. 68.2±10.6 mm GMA-MS and 52.3±3.6 mm Ni-Cu WF, mean ± SD) (**Figure 4A**). ESR was also carried out with the particles incubated for 5 minutes at 37°C with RAW 264.7 cells. The goal was to examine whether these inflammatory cells produce free radicals as a quick, oxidative burst reaction to the welding fumes. Compared to PBS-treated control cells, all 3 welding fumes produced peaks in the spectra (93.7±17.2 mm GMA-MS, 79.5±16.5 mm GMA-SS, 56.8±8.0 mm Ni-Cu WF, mean ± SD) (**Figure 4B**). However, both the GMA-MS and GMA-SS samples caused significantly greater hydroxyl radical production compared to Ni-Cu WF. To confirm the role of endogenous H_2_O_2_ from cells as the source by which the welding fumes generate hydroxyl radicals, additional samples were run in the presence of catalase, which is a strong H_2_O_2_ scavenger. This addition abolished peaks with all three welding fume samples, as in previous ESR studies with metal-containing particles [20], [22], [31].

### Intracellular ROS production by welding fumes

**Figure 5:**
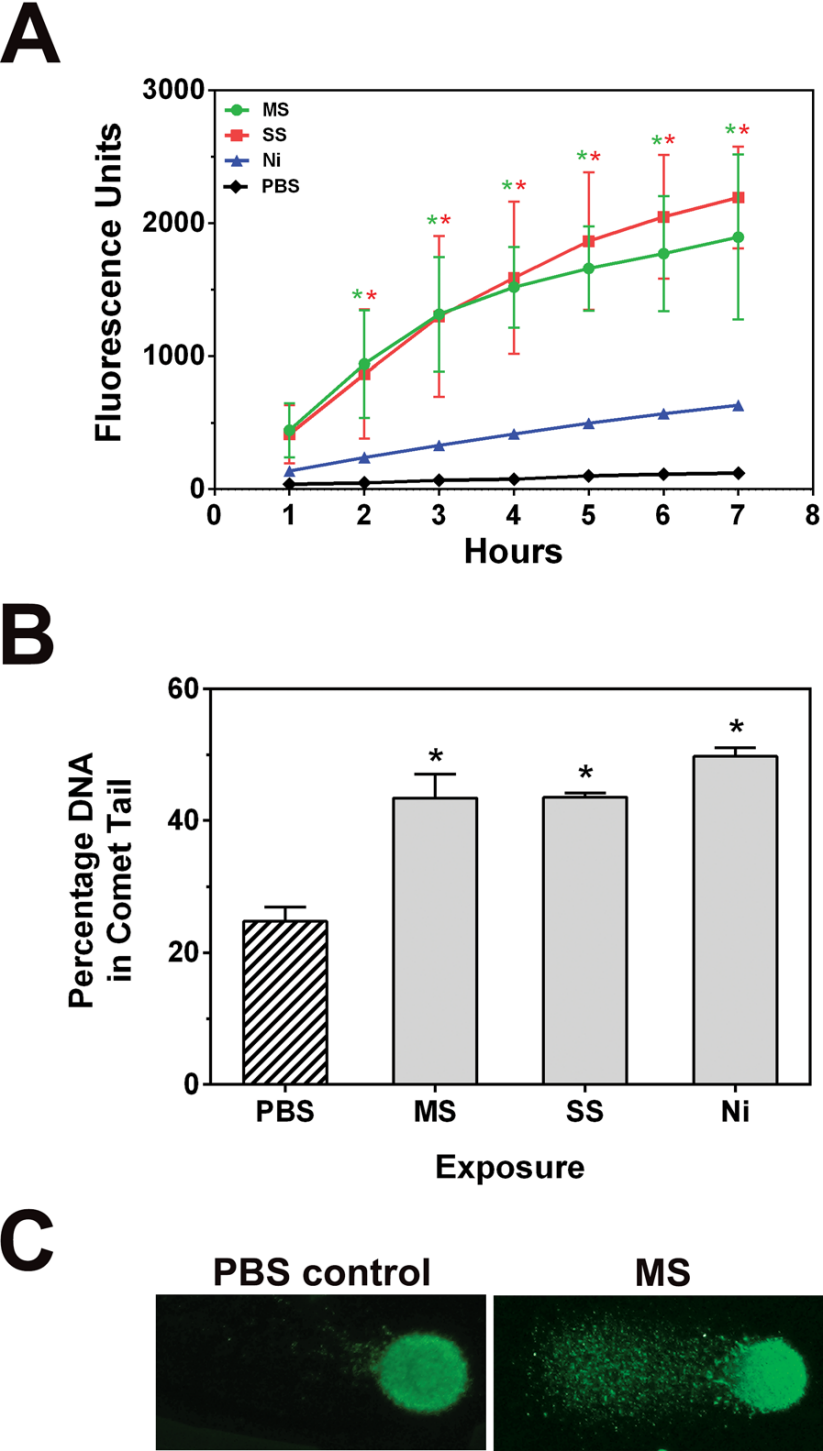
Intracellular ROS production in cells following treatment with welding fumes. **A.** RAW 264.7 cells were pre-treated with DCFH-DA, exposed to 50 µg/ml suspensions of welding fumes, and plates were read each hour to measure intracellular ROS. Error bars represent the mean ± SD (n = 3). *, p<0.05 compared to PBS controls at that time point. **B.** A comet assay was used to examine DNA damage. Cells were treated with 50 µg/ml suspensions of welding fumes for 3 h, washed and scraped into PBS, added to glass slides with agarose, then lysed and subjected to electrophoresis. SYBR green was added to stain double-stranded DNA. Images were acquired using fluorescence microscopy and a 40x objective. Experiments were performed in duplicate and comets were measured by comparing the corrected nuclear region fluorescence to the corrected total cell fluorescence. This was converted to a percentage to indicate DNA damage. Error bars represent the mean ± SD. *, p<0.05 compared to PBS controls. **C.** Representative images of comets.

To further explore the influence of the welding fumes on ROS production in cells, an intracellular ROS assay was used. Utilizing DCFH-DA (2′,7′-dichlorodihydrofluorescein diacetate) as a fluorescent probe for ROS within cells, we were able to monitor changes in fluorescence over a time course of 7 hours. Only the 50 µg/ml dose was used for these experiments, because the 250 µg/ml dose appears to be cytotoxic with all three samples (**Figures 1** **–** **3**) and mostly viable cells were needed for ROS measurements. **Figure 5A** shows that GMA-MS and GMA-SS induced strong intracellular ROS production starting at the 2 hour time point and this continued to increase over several hours, whereas the Ni-Cu WF only induced minimal ROS over the time course. This agrees with the ESR data, in which Ni-Cu WF was less capable of producing free radicals compared to the GMA-MS/SS fumes.

An alkaline comet assay was also performed to determine whether the intracellular ROS from fume exposures caused oxidative damage to nuclear DNA. The comet assay has been long used to visualize DNA strand breakage as an indicator of genotoxic insult [32], [33]. Our findings indicated that cells treated for 3 hours with 50 µg/ml welding fumes had significant DNA damage with all exposures (43.5±3.6% GMA-MS, 43.6±0.6% GMA-SS, and 49.7±1.3% Ni-Cu WF vs. 24.8±2.2% PBS, mean ± SD) (**Figure 5B**). This suggests that the persistence or redox cycling of ROS within welding fume-exposed cells can lead to downstream oxidative damage.

### Welding fumes impair phagocytic function of RAW 264.7 cells

**Figure 6:**
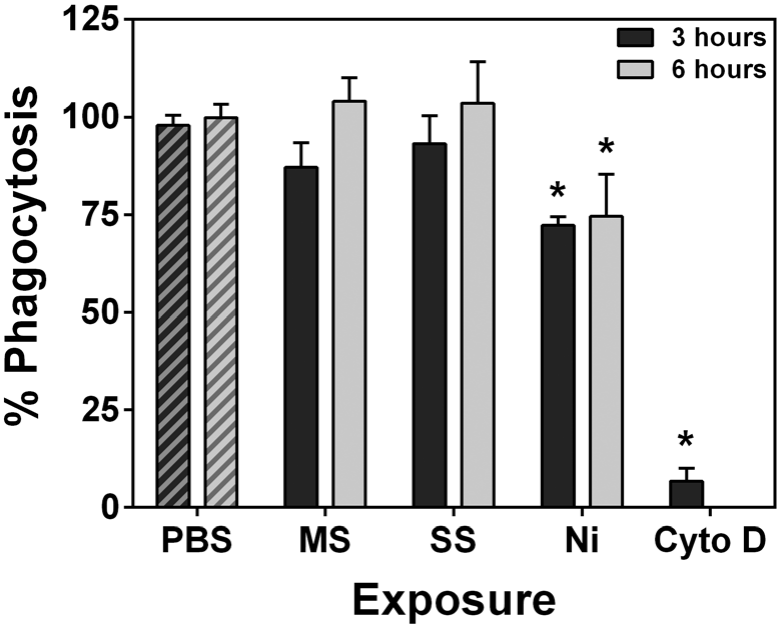
Pre-incubation with Ni-Cu WF impairs phagocytosis. RAW 264.7 cells were treated with 50 µg/ml welding fume suspensions for 3 or 6 h or cytochalasin D (Cyto D) for 3 h. Cells were washed, pHrodo Red *E. coli* BioParticles were added for 2 h, and plates were read to measure changes in fluorescence. Error bars represent the mean ± SD (n = 4). *, p<0.05 compared to PBS controls.

Macrophages infiltrate the lung following welding fume inhalation and appear to play a role in the pulmonary clearance of these particles [34]–[36]. However, it has also been shown that GMA welding fumes can negatively affect macrophage function [7], [37]. Thus, we examined the ability of RAW 264.7 cells to properly phagocytize *E. coli* particles following 3 or 6 hour welding fume exposures at 50 µg/ml. The shorter time points were chosen, because exposure to Ni-Cu WF for 24 hours reduced cell viability at this lower dose (**Figures 1** **,** **2**), and we wanted the cells to be viable in order to observe changes in function prior to cytotoxicity. **Figure 6** shows that only pre-treatment with Ni-Cu WF significantly impaired subsequent phagocytosis of a pathogen-based particle compared to PBS controls (72.3±2.1% and 74.5±10.8% at 3 and 6 hours, respectively, mean ± SD) while GMA-MS and GMA-SS had no effect at these time points and at this concentration. These results suggest that perhaps pre-uptake of GMA-MS and GMA-SS cause cells to produce more ROS, whereas Ni-Cu WF negatively affects macrophage function by reducing the phagocytic ability prior to cytotoxicity. We hypothesize that whatever negative effect these particles had on the RAW 264.7 cells’ ability to phagocytose bacteria was already occurring by the 3 hour time point. Thus, at 6 hours and perhaps later times, the cells would still not be able to properly take up the *E. coli* BioParicles.

### Cytokine production from welding fumes

**Figure 7:**
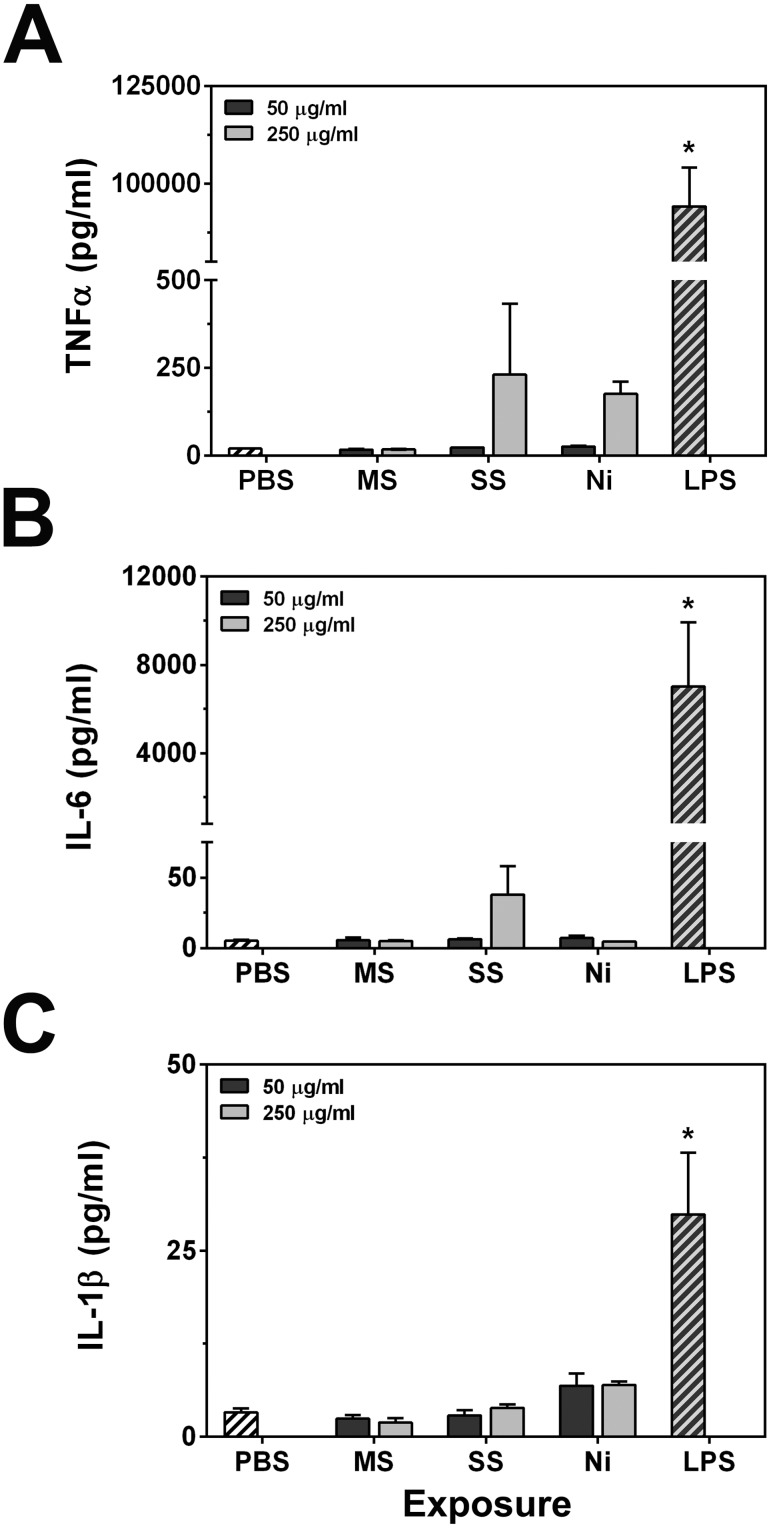
Welding fumes do not induce pro-inflammatory cytokine production. RAW 264.7 cells were treated with welding fume suspensions or 1 µg/ml LPS for 24 h. Media were collected, and ELISAs were run to measure levels of TNFα (**A**), IL-6 (**B**) and IL-1β (**C**). Error bars represent the mean ± SD (n = 3). *, p<0.05 compared to PBS controls.

Based on the above results showing ROS production and previous *in vivo* studies that have shown GMA welding fumes to cause pulmonary inflammation [34], [37]–[39], we hypothesized that cytokine production may occur in cells following welding fume exposures. Unprimed RAW 264.7 cells were exposed to welding fumes for 24 hours and ELISAs were used to measure TNFα, IL-6, and IL-1β (**Figure 7**). Although GMA-SS treatment appeared to induce some cytokine production (TNFα and IL-6 at 250 µg/ml after 24 hours exposure), the results were not statistically significant from PBS controls. The cellular conditions at 24 hours included at least some portion of the cells undergoing death at the higher dose (**Figures 1** **and** **2**), so it is possible that this triggered some mild production and release of cytokines. However, in general, it appears that the welding fumes are not able to induce robust cytokine production from unprimed macrophages *in vitro*.

## Discussion

Previous studies of fumes generated from welding processes have demonstrated their toxic potential both *in vitro* and *in vivo*. In animal studies, SS fumes appear to be more pneumotoxic than MS fumes [40]–[43], and this enhanced injury potential is thought to be due to differences in metal constituents. Specifically, the presence of potentially toxic metals, such as Cr(VI), Mn, Ni, and Fe have been speculated to be involved in SS welding fumes’ toxicity. For example, Mn from welding fumes has been implicated in neurotoxicity [9]–[11], while Cr-containing SS fumes have been shown to act as a lung tumor promoter [6], [44]. Thus, there has been an effort to develop welding consumables that contain less of these metals while maintaining favorable weldability properties. Although the newly-developed Ni-Cu WF contains Ni (13.4%) and other toxic metals, it has considerably less Cr, Mn, and Fe than GMA-SS (0.47%, 0.46%, and 1.65% in Ni-Cu WF vs. 20.3%, 13.8%, and 57.2% in GMA-SS, respectively) and is being proposed as a potentially less-toxic alternative to SS consumables. However, no toxicological studies have been carried out with this material. Thus, the current study aimed to evaluate the potential adverse effects of this welding fume and compare those results with previously-studied GMA-MS and GMA-SS fumes to determine whether a Ni-Cu-based consumable may be less hazardous to welders.

The metal profiles in **Table 1** show that Fe makes up the majority of GMA-MS and GMA-SS (82.8% and 57.2%, respectively), while Ni-Cu WF contains very little Fe (1.65%). Transition metals such as Fe and Cr(VI) participate in Fenton reduction/oxidation cycling and are known to mediate ROS production [45], [46]. Therefore, our findings that GMA-MS and GMA-SS can produce significantly more ROS than Ni-Cu WF (**Figures 4** **and** **5**) are likely attributable to the high amounts of Fe present in the GMA fumes. Even though Ni-Cu WF has very low levels of Fe and Cr(VI), its ability to generate some ROS in our assays is not surprising, as this fume contains other redox-active metals, such as Ni, Cu, titanium, and zinc. However, at first glance, these findings make the Ni-Cu-based welding material more appealing than steel-based materials, as the oxidative potential of metal-containing particles is a major concern for pulmonary toxicity in many studies of health effects. For example, it has been shown that soluble transition metals in particulate matter such as residual oil fly ash [47] and diesel exhaust particles [48], [49] are likely responsible for the pulmonary injury they induce due to their ability to produce ROS and pro-inflammatory mediators. Factors other than excess ROS must be at least partially responsible for the viability loss at the higher dose (250 µg/ml), as pre-treatment with an antioxidant did not elicit a rescue effect for cells treated with the welding fumes (**Figure 2**).

Regardless of the diminished ROS production, Ni-Cu WF is more overtly toxic to RAW 264.7 cells than GMA-MS and GMA-SS. This conclusion was drawn from the fact that only Ni-Cu WF was able to significantly reduce the number of live cells and indices of mitochondrial function/bioenergetics at the lower dose of 50 µg/ml (**Figures 1** **–** **3**). The toxic effects of this welding fume are likely to be at least partially due to its Ni content, as others have shown Ni-containing particles to be cytotoxic to cultured lung epithelial [50] and macrophage [51] cells among others. However, GMA-SS has a similar amount of Ni present, so our findings cannot be explained by Ni content alone. It has been well-documented that Cu-containing [52], [53] and titanium-containing [54], [55] nanoparticles also induce damage in cultured cells, so we hypothesize that it is the combination of multiple toxic metals that cause Ni-Cu WF’s enhanced cytotoxicity.

In the comet assay, we anticipated greater DNA “tails” with GMA-MS and GMA-SS over Ni-Cu WF, because of their enhanced ability to induce ROS production. However, Ni-Cu WF appears to damage DNA to an equal or even greater extent in the cultured macrophages. This finding may due to early apoptosis or necrosis in addition to any ROS production, since cell death induces double strand breaks that contribute to comet tails [56], [57]. Additionally, several studies have demonstrated (some through the comet assay) that Ni compounds are carcinogenic as a result of their ability to potently induce oxidative DNA damage [58]–[60]. Thus, DNA damage caused by Ni-Cu WF exposures could potentially precede any carcinogenic effects that these particles may have.

The mitochondrial function assay was helpful in providing more detail about specific parameters of bioenergetics that were altered following welding fume exposures. Dranka *et al*. (2010) used this system to analyze the effects of nitric oxide and ROS on mitochondrial function in endothelial cells and found that the reserve capacity plays a crucial role in how cells respond to oxidative stress. When a redox cycling agent was added to cells, the basal OCR was stimulated and the calculated reserve capacity was found to be significantly reduced [27]. This ROS-induced drop in spare capacity was also observed by another group, when non-toxic concentrations of H_2_O_2_ were added to cultured endothelial cells [28]. We had similar findings with GMA-MS and GMA-SS: elevated basal OCR traces (**Figure 3A**) and slightly reduced spare capacities with 50 µg/ml exposures. This suggests that although the cells are mostly viable with these exposures, the mitochondrial function may be altered due to excess ROS production. It could also indicate a breakdown in the electron transport chain, which would account for some of the excess ROS production. Sriram *et al.* (2010) reported that neuroinflammation and neurotoxicity in rats exposed to GMA-MS was due to mitochondrial dysfunction [9], so it is possible that these effects would be observed in neuronal cells as well. They attributed the mitochondrial dysfunction, oxidative stress, and neuronal cell death to the Mn content of the welding fumes. The spare capacity reduction was more drastic with Ni-Cu WF at this dose, which was likely due to fewer healthy, live cells versus subtle mitochondrial dysfunction that can be ascertained with the GMA-MS and GMA-SS data.

Our findings that GMA-MS and GMA-SS are very similar in terms of their cytotoxicity and ROS production are inconsistent with previous data showing GMA-SS to be more toxic than GMA-MS. A potential reason for this discrepancy could be that these fumes were not freshly generated, as they were in several previous reports. In fact, our group has seen significant differences in GMA-SS reactivity in RAW 264.7 cells [16] and in rats [41] depending on whether they were fresh or aged samples (i.e., collected 1 or more days before exposure). This is thought to be due to the presence of short- and long-lived ROS present on the surface of freshly generated fumes, which decays with time [61], [62].

The welding fume exposures clearly alter RAW 264.7 function, whether through excessive ROS production or impaired bioenergetics. However, because our *in vitro* model utilizes these monocyte macrophages, we were also interested in any effects the fumes would have on phagocytosis. Studies of particulate matter have demonstrated that various exposures (i.e., diesel exhaust, ultrafine carbon, titanium dioxide, and concentrated ambient particles) impair the ability of cultured macrophages to properly phagocytize and kill pathogens [63]–[66]. The same has been shown *in vitro* with certain welding fumes [67]. Further, altered lung defense responses and slowed pulmonary clearance of bacterial pathogens have been seen following pulmonary exposure with welding fumes [29], [37], [68]. These *in vivo* studies implicate Cr(VI) as the metal constituent that is mostly responsible for compromised pulmonary clearance, but GMA-SS, which contains the most Cr(VI), did not impair phagocytosis in our study (**Figure 6**). This is likely due to the fact that our study only examined the response of a single cell type, in contrast to animal models which involve signaling and phagocytosis by multiple cell types within the lung. However, because exposure to Ni-Cu WF can reduce phagocytosis in RAW 264.7 cells, it is reasonable to hypothesize that this fume would also be capable of altering host lung defenses in animal models.

The present study is the first, to our knowledge, to evaluate the toxicity of fumes generated from a new Ni-Cu-based welding material. In general, these particles appear to be cytotoxic to cultured macrophages, causing DNA damage and impairing phagocytosis. It is clear from our results that changing the metal contents of the welding material influences the toxic response by cells, and that minimizing Cr(VI) and Mn content does not reduce its toxic potential if other metal components are capable of inducing cell damage and altering cell function. Given the strong epidemiologic evidence of the hazards of welding fume exposures, less-toxic welding consumables are highly sought after. However, our results indicate that this new material may not be a suitably safer alternative to currently used consumables.
